# Tenecteplase in acute ischemic stroke: an updated meta-analysis

**DOI:** 10.3389/fneur.2026.1921538

**Published:** 2026-09-15

**Authors:** Hussain Almohammed, Ahmed Salah Morad, Bushra Wadi Bin Saddiq, Fatimah Ibrahim Almuhaysin, Bayan Mohammed Khair Al Zoabi, Wejdan Ahmed Aldawsari, Tanveer Nidal Khan, Husna Irfan Thalib, Zainab Alabdi

**Affiliations:** 1 College of Medicine, King Faisal University, Al Ahsa, Saudi Arabia; 2 General Medicine Practice, Batterjee Medical College, Jeddah, Saudi Arabia; 3 College of Medicine, Tabuk University, Tabuk, Saudi Arabia; 4 Neurology Department, King Fahad Hospital, Al Ahsa, Saudi Arabia

**Keywords:** alteplase, door-to-needle, mortality, stroke, tenecteplase

## Abstract

**Introduction:**

Tenecteplase (TNK) has emerged as a promising alternative to alteplase (tPA) for intravenous thrombolysis in acute ischemic stroke (AIS). This study aimed to evaluate the efficacy and safety of TNK compared with tPA.

**Methods:**

Following the PRISMA 2020 guidelines, PubMed, Web of Science, Cochrane Central, EBSCO, and Scopus were systematically searched from inception through January 2026 to identify randomized controlled trials (RCTs) and observational studies comparing TNK with tPA in adults with AIS. Effect estimates were pooled using fixed- or random-effects models according to the degree of heterogeneity and were reported as risk ratios (RRs), odds ratios (ORs), or mean differences (MDs) with 95% confidence intervals (CIs).

**Results:**

Twenty-four studies (6 RCTs and 18 observational studies; total *n* = 20,592) involving patients with AIS were included. Compared with tPA, TNK was associated with a greater likelihood of excellent functional recovery (mRS 0–1) at 90 days (RR = 1.08; 95% CI: 1.01–1.15; *p* = 0.02) and a shorter door-to-needle time by an average of 4 min (MD = −4.20; 95% CI: −7.77 to −0.64; *p* = 0.02). Mortality was lower among patients treated with TNK (OR = 0.83; 95% CI: 0.73–0.94; *p* = 0.004). However, subgroup analyses restricted to RCTs showed no significant difference in mortality between the TNK and tPA groups (OR = 0.99; 95% CI: 0.82–1.21; *p* = 0.95).

**Conclusion:**

TNK provides efficacy and safety profiles comparable to those of tPA while offering a greater likelihood of excellent functional recovery, faster treatment initiation, and lower mortality in observational studies. Further high-quality RCTs are warranted to confirm these findings.

**Systematic review registration:**

CRD420251030320.

## 1 Introduction

Ischemic stroke is a significant cause of long-term disability and mortality, accounting for 62.4% of all strokes worldwide (1). Intravenous thrombolysis using alteplase (0.9 mg/kg, to a maximum of 90 mg) remains the gold standard for the treatment of acute ischemic stroke (AIS) (2). Its ability to dissolve arterial occlusions was first tested in ischemic heart disease patients in 1984, and it showed successful coronary artery opening in 75% of patients (3). Despite its undisputed efficacy, alteplase has a narrow 4.5-h treatment window from stroke onset and suboptimal recanalization rates (2). While alteplase was the only approved thrombolytic for AIS by the U. S. Food and Drug Administration (FDA) since 1996, tenecteplase (TNK) has become the second agent to be approved after nearly 30 years (4).

Studies suggest that TNK may outperform tPA in key aspects. Alteplase has a short half-life of 4 min and requires a 60-min-long infusion following bolus administration, in contrast to TNK, which has a longer half-life of 20 min (5–7). Moreover, TNK has more fibrin-specific properties, which are very helpful in reducing systemic effects and fibrinogen depletion (8). Furthermore, the optimal dose of TNK is 0.25 mg/kg for patients with AIS. In contrast, higher doses, such as TNK 0.40 mg/kg, should be avoided in patients with AIS due to increased risk of symptomatic intracranial hemorrhage (sICH) (9).

The EXTEND-IA TNK trial found higher early reperfusion rates in patients who took tenecteplase than those on alteplase (22% vs. 10%, *p* = 0.002) before thrombectomy (10). However, NOR-TEST did not find a notable difference in clinical outcome, frequency of sICH, or mortality at 7 days, yet the TNK group showed a higher all-cause mortality at 90 days (26.3% vs. 9.1%) (11), which underscores the need for investigating differences in efficacy and safety based on patient selection, dosing, and real-world applicability.

This systematic review and meta-analysis evaluated data from randomized controlled trials (RCTs) and observational studies to compare the efficacy and safety of TNK with alteplase in the setting of acute ischemic stroke, with a particular focus on whether TNK is non-inferior to alteplase. The findings may help inform the use of TNK as an alternative to alteplase in acute ischemic stroke.

## 2 Methodology

### 2.1 Protocol approval and registration

This systematic review and meta-analysis followed the recommendations of the Preferred Reporting Items for Systematic Reviews and Meta-Analyses (PRISMA) 2020 guideline checklist (12). The protocol for the current study was registered in PROSPERO with the following ID number: CRD420251030320. Due to the nature of this study, which involved secondary analysis of previously published data, ethical approval and informed consent were not required.

### 2.2 Search strategy

The electronic databases PubMed, Web of Science, Cochrane Central, EBSCO, and Scopus were systematically searched to extract all relevant articles published until January 2026. Across all databases, the search strategy was developed using predefined keywords encompassing stroke-related terms (minor stroke, transient ischemic attack, cerebral stroke, cerebral infarction, cerebrovascular disease, cerebral ischemia, cerebrovascular disorders, cerebrovascular accident, brain ischemia, acute stroke, brain infarction, light stroke, mild stroke, silent stroke), thrombolytic agents (tenecteplase, TNK-tPA, TNK, TNKase, rhTNK-tPA), comparator terms (alteplase, rt-PA, tissue plasminogen activator, plasminogen activator tissue type), and methodological descriptors (randomized controlled trial, controlled clinical trial, randomized, placebo-controlled, drug therapy, clinical trial, random assignment, trial, study groups, cohort study, retrospective study, prospective study). The exact Boolean search strings used for each database are provided in Supplementary Table S1. In addition, the reference lists of all included studies were manually screened to identify any relevant studies not captured by electronic search. No gray literature sources or trial registries were consulted.

### 2.3 Study selection criteria

The studies were eligible for inclusion if they were published in English and included patients aged ≥18 years who were diagnosed with AIS. Eligible study designs included RCTs and cohort studies. Studies must have completed their trials and were required to report at least one neurological outcome, such as NIHSS before and after 24 h or mRS at 90 days. In addition, studies had to report at least one predefined efficacy or safety outcome to be eligible for inclusion. Studies were excluded if they involved patients under 18 years of age, patients without a confirmed diagnosis of AIS, patients who did not receive TNK, or studies that did not directly compare TNK with alteplase (single-arm studies). Additionally, studies not reporting the required neurological outcomes, incomplete or ongoing clinical trials without outcome data, non-english language articles, case reports, case–control studies, cross-sectional studies, systematic reviews, literature reviews, editorials, conference abstracts and animal studies were excluded. Studies that did not have available data to get effect estimates were excluded.

### 2.4 Screening

Two reviewers (F.A. and B.A.) independently screened all articles retrieved through the search process. Initial screening based on titles and abstracts was conducted using Rayyan software (13). Articles that met the inclusion criteria proceeded to full-text screening, which was also performed independently by the same reviewers. Any disagreements were discussed and resolved through consensus. When a consensus could not be reached, a third reviewer (H.A.) was consulted to resolve the disagreement.

### 2.5 Data extraction

Two reviewers (H.A. and F.A.) independently extracted data from each study. Extracted information included study title, authors, publication year, journal, country of origin, study design, number of participants, demographics, TNK dosage, timing of administration, and site of vessel occlusion.

### 2.6 Risk of Bias assessment and certainty of evidence assessment

Risk of bias was assessed using the Cochrane risk of bias tool 2 (ROB2) for randomized controlled trials and the Newcastle-Ottawa Scale (NOS) for cohort studies (14, 15). The Grading of Recommendations, Assessment, Development and Evaluations (GRADE) approach was used to assess the certainty of the evidence. The quality of evidence was classified as high, moderate, low, or very low. All procedures were conducted using GRADEpro software.

### 2.7 Outcome measures

The primary efficacy outcomes assessed in this review were the improvement in NIHSS score at 24 h, improvement of mRS at 3 months, door-to-needle time (DTN), and onset-to-needle time (OTN). The secondary outcomes concerned the safety of both interventions, such as sICH, parenchymal hematoma, mortality rate, and angioedema. These outcomes were chosen to comprehensively evaluate the functional recovery and potential adverse events associated with thrombolytic treatment in patients with AIS, thereby providing a detailed comparison between tenecteplase and alteplase. Comprehensive definitions of all outcomes are presented in Supplementary Table S2.

### 2.8 Data analysis

This meta-analysis utilized findings from the studies included in the search to determine the efficacy and safety of tenecteplase compared to alteplase in patients diagnosed with AIS. Statistical analysis was conducted using the RevMan Manager (RevMan) Version 5.4 software, which aided in pooling the study results and evaluating overall treatment effects (16). Heterogeneity across studies was examined using the Cochrane-based Q statistic and I^2^ test, which determined the type of model to be further used in the analysis. A random-effects model was applied when substantial heterogeneity was present (*p* < 0.05, I^2^ > 50%). In cases where heterogeneity was minimal (I^2^ < 50%, *p* > 0.05), a fixed-effects model was used instead. Standardized mean differences (SMD) for continuous outcomes and odds ratios (OR) for dichotomous variables were used to express the effects of treatment; each had a 95% confidence interval (CI). *p*-values below 0.05 were regarded as statistically significant. Subgroup analysis was performed to explore potential reasons for the high heterogeneity present in some of the outcomes. The subgroup was considered valid if at least two studies fit its criteria. The studies were subgrouped based on the implemented study design and the TNK dose used. Sensitivity analysis was also conducted in general to discern if exclusion of any of the studies led to a significant change in the results. Additionally, due to the availability of an adequate number of studies, a funnel plot could be charted to assess the publication bias.

## 3 Results

### 3.1 Search results

Figure 1 illustrates the PRISMA flow chart depicting the study inclusion steps in our review. Using our search strategy, we retrieved 3,503 studies from five databases. Of them, 589 duplicates were identified and removed. Further screening based on title and abstract excluded 2,817 studies. After assessing the eligibility of the remaining 90 studies based on the full text, 66 were excluded. Finally, 24 studies were included in this systematic review and meta-analysis (7, 17–39).

**Figure 1:**
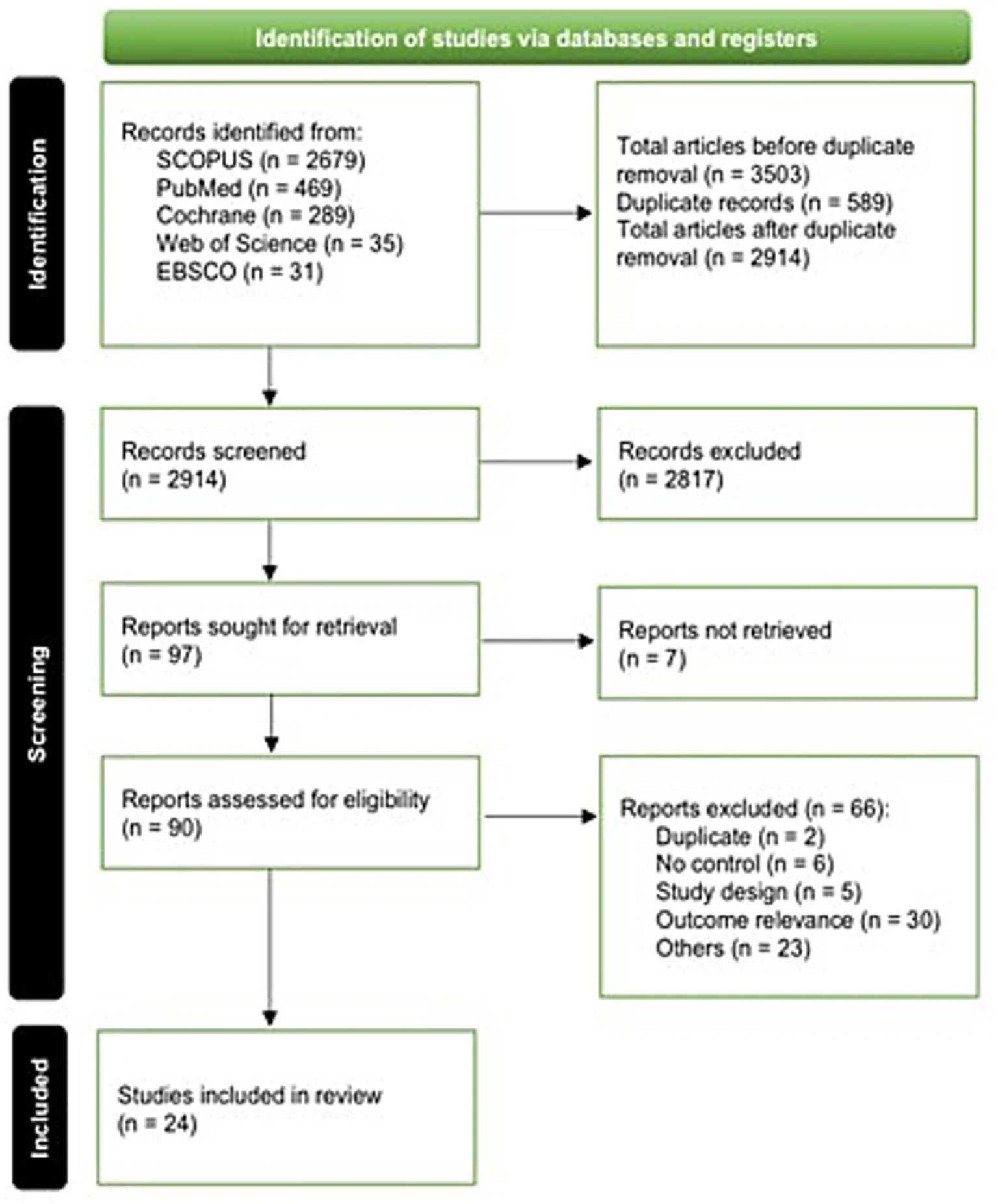
PRISMA flowchart.

### 3.2 Summary of study characteristics

Table 1 presents the baseline characteristics of the studies investigating. A total of 24 studies were included, comprising a mix of RCTs and cohort studies. These studies involved 20,592 patients, with 10,421 patients in the TNK group and 10,171 in the tPA group. The mean age of patients was 68.7 ± 14.5 years in the TNK group and 68.5 ± 14.7 years in the tPA group. Gender distribution was similar across both arms, with males comprising 57.8% of the TNK group and 57.6% of the tPA group. Sites of arterial occlusion varied across studies, most commonly involving the internal carotid artery (ICA), middle cerebral artery (MCA1, MCA2, MCA3), basilar artery, and tandem occlusions. In terms of dosing, TNK was most frequently administered at 0.25 mg/kg, while tPA was given at the standard 0.9 mg/kg. Some studies used capped doses for TNK (e.g., 0.2 mg/kg with a 20 mg max) (30, 33, 35), and one study used a slightly higher Tenecteplase dose of 0.4 mg/kg (37). Both drugs were typically given as single boluses in the acute stroke setting.

**Table 1:** Study characteristics.

Study ID	Country	Design	Number of participants			Mean age of participants (± SD)*		Gender (*n* (%); M/F)		Mean daily dose		Follow-up	Site of occlusion	
			TNK	tPA	Total	TNK	tPA	TNK	tPA	TNK	tPA		TNK	tPA
Henderson 2024 (17)	USA	Observational	50	50	100	75 ± 16.7	78.5 ± 15.6	M: 23 (46%) F: 27 (54%)	M: 25 (50%) F: 25 (50%)	0.25 mg/Kg	0.9 mg/Kg	N/A	ICA, MCA 1, MCA 2	ICA, MCA 1, MCA 3
Lo 2024 (18)	Hong Kong	Observational	148	138	286	81 ± 13.7	79 ± 14.1	M: 65 (43.9%) F: 83 (56.1%)	M: 70 (50.7%) F: 68 (49.3%)	0.25 mg/Kg	0.9 mg/Kg	3 months	N/A	N/A
Hendrix 2024 (19)	USA	Observational	309	326	635	70 ± 12.6	69 ± 15.6	M: 152 (49.2%) F: 157 (50.8%)	M: 168 (51.5%) F:158 (48.5%)	0.25 mg/Kg	0.9 mg/Kg	3 months	ICA, MCA 1, MCA 2, Basilar artery, Tandem occlusion of ICA and MCA	ICA, MCA 1, MCA 2, Basilar artery, Tandem occlusion of ICA and MCA
Karamchandani 2024 (20)	USA	Observational	109	124	233	66.0 ± 17.7	67.8 ± 15.6	M: 49 (45.0%) F: 60 (55.0%)	M: 67 (54.0%) F: 57 (46.0%)	N/A	N/A	3 months	ICA, MCA 1, MCA 2	ICA, MCA 1, MCA 3
Muir 2024 (21)	UK	RCT	885	892	1777	70.4 ± 12.5	70.4 ± 13.4	M: 533 (60%) F: 351 (40%)	M: 527 (59%) F: 365 (41%)	0.25 mg/Kg	0.9 mg/Kg	3 months	MCA 2	MCA 3
Zarzour 2024 (22)	France	Observational	250	881	1,131	75 ± 17.0	74 ± 14.8	M: 114 (45.4%) F: 136 (54.6%)	M: 422 (47.9%) F: 459 (52.1%)	0.25 mg/Kg	0.9 mg/Kg	3 months	ICA, MCA 1, MCA 2, Basilar artery, Tandem occlusion of ICA and MCA	ICA, MCA 1, MCA 2, Basilar artery, Tandem occlusion of ICA and MCA
Figurelle 2025 (23)	USA	Observational	2,305	3,337	5,642	66.70 ± 15.76	65.63 ± 15.71	M: 1112 (48.65%) F: 1183 (51.35%)	M: 1656 (49.61%) F: 1681 (50.39%)	N/A	N/A	N/A	N/A	N/A
Liu 2024 (24)	China	Observational	1,113	2,360	3,473	66 ± 10.7	68 ± 11.9	M: 713 (64.06%) F: 400 (35.94%)	M: 1567 (66.4%) F: 793 (33.6%)	0.25 mg/Kg	0.9 mg/Kg	3 months ±7 days	N/A	N/A
Dutta 2024 (25)	India	Observational	71	83	154	60 ± 39.3	68 ± 44.4	M: 41 (57.7%) F: 30 (42.3%)	M: 48 (57.8%) F: 35 (42.2%)	0.25 mg/Kg	0.9 mg/Kg	3 months	ICA, MCA 1, MCA 2, Basilar artery, Tandem occlusion of ICA and MCA	ICA, MCA 1, MCA 2, Basilar artery, Tandem occlusion of ICA and MCA
Yao 2024 (26)	China	Observational	120	144	264	67.75 ± 12.76*	67.75 ± 12.76	M: 91 (75.8%) F: 29 (24.2%)	M: 98 (68.1%) F: 46 (31.9%)	0.25 mg/Kg	0.9 mg/Kg	N/A	N/A	N/A
Hendrix 2022 (27)	USA	Observational	51	97	148	72 ± 11.9	74 ± 15.6	M: 25 (49.0%) F: 26 (51.0%)	M: 51 (52.6%) F: 46 (47.4%)	N/A	N/A	3 months	ICA, MCA 1, MCA 2, Basilar artery, Tandem occlusion of ICA and MCA, PCA, VA	ICA, MCA 1, MCA 2, Basilar artery, Tandem occlusion of ICA and MCA, PCA, ACA, VA
Wang 2023 (7)	China	RCT	710	707	1,417	67 ± 11.1	65 ± 10.4	M: 492 (69%) F: 218 (31%)	M: 479 (68%) F: 228 (32%)	0.25 mg/Kg	0.9 mg/Kg	3 months	N/A	N/A
Mohan 2023 (28)	India	Observational	57	103	160	57 ± 12	54 ± 15	M: 43 (75.44%) F: 14 (24.56%)	M: 62 (60.20%) F: 41 (39.80%)	0.25 mg/Kg	0.9 mg/Kg	3 months	N/A	N/A
Teivane 2022 (29)	Latvia	Observational	45	139	184	68.4 ± 13.5	73.0 ± 11.4	M: 21 (46.7%) F: 24 (53.3%)	M: 68 (48.9%) F: 71 (51.1%)	0.25 mg/Kg	0.9 mg/Kg	3 months	N/A	N/A
Dhar 2022 (30)	India	Observational	23	19	45	60 ± 42.9	60 ± 30.4	M: 17 (73.9%) F: 6 (26.1%)	M: 11 (57.9%) F: 8 (42.1%)	0.2 mg/kg	0.9 mg/kg	3 months	ICA, MCA 1, Basilar artery, Tandem occlusion of ICA and MCA	ICA, MCA 1, Basilar artery, Tandem occlusion of ICA and MCA
Tsivgoulis 2022 (31)	Multinational	Observational	331	797	1,128	70 ± 16.3	70 ± 14.1	M: 185 (56%) F: 146 (44%)	M: 446 (56%) F: 351 (44%)	0.25 mg/Kg	0.9 mg/kg	3 months	N/A	N/A
Estella 2022 (32)	Spain	Observational	20	80	100	73 ± 7.0	74.5 ± 11.7	M: 15 (80%) F: 4 (20%)	M: 40 (50%) F: 40 (50%)	0.25 mg/Kg	0.9 mg/kg	3 months	N/A	N/A
Georgea 2021 (33)	India	Observational	61	29	90	64.3 ± 11.3	63.2 ± 12.01	M: 37 (60.6%) F: 24 (39.3%)	M: 19 (65.5%) F: 10 (34.4%)	0.2 mg/kg	0.9 mg/kg	3 months	N/A	N/A
Psychogios 2021 (34)	Greece	Observational	19	39	58	69 ± 20*	70 ± 10.4*	M: 11 (57.9%) F: 8 (42.1%)	M: 14 (35.8%) F: 25 (64.2%)	0.25 mg/Kg	0.9 mg/kg	3 months	ICA, MCA 1, MCA 2, Basilar artery	ICA, MCA 1, MCA 2, Basilar artery
Sundar 2019 (35)	India	Observational	55	65	120	66.6 ± 8.72	62.5 ± 9.22	M: 43 (78.2%) F: 12 (21.8%)	M: 40 (61.5%) F: 25 (38.5%)	0.2 mg/kg	0.9 mg/kg	3 months	N/A	N/A
Campbell 2018 (36)	Australia & New Zealand	RCT	101	101	202	70.4 ± 15.1	71.9 ± 13.7	M: 58 (57%) F: 43 (43%)	M: 52 (51%) F: 49 (49%)	0.25 mg/Kg	0.9 mg/kg	3 months	N/A	N/A
Logallo 2017 (37)	Norway	RCT	549	551	1,100	77 ± 11.1	77 ± 11.1	M: 321 (58%) F: 228 (42%)	M: 339 (62%) F: 212 (38%)	0.4 mg/kg	0.9 mg/kg	3 months	N/A	N/A
Meng 2024 (38)	China	RCT	732	733	1,465	66 ± 11.1	65 ± 11.9	M: 517 (70.6%) F: 215 (29.4%)	M: 502 (68.5%) F: 231 (31.5%)	0.25 mg/Kg	0.9 mg/kg	3 months	N/A	N/A
Parsons 2024 (39)	Multinational	RCT	339	341	680	75 ± 12.6	73 ± 13.3	M: 202 (60%) F: 137 (40%)	M: 218 (64%) F: 123 (36%)	0.25 mg/Kg	0.9 mg/kg	3 months	N/A	N/A

TNK; Tenecteplase, tPA; Tissue plasminogen activator, SD; standard deviation, RCT; Randomized controlled trial, ICA; Internal carotid artery, MCA; Middle cerebral artery. *Estimated mean and standard deviations.

### 3.3 Quality assessment

Among the six RCTs, two were judged to have a low overall risk of bias, while four were rated as having an unclear risk. The uncertainty in these four trials stemmed mainly from limited reporting within key ROB2 domains. In Wang (7), Logallo (37), Meng (38), and Parsons (39), concerns were noted in the deviations from intended interventions domain due to insufficient details on adherence to treatment protocols. Additionally, Logallo (37) showed concerns in the outcome measurement domain because assessor blinding was not clearly described. Other than this, all RCTs demonstrated low risk regarding missing outcome data, outcome measurement except (37), and selective reporting, indicating consistent and unbiased assessment (Figure 2). For the 18 retrospective and prospective cohort studies. Sixteen studies were rated as low risk, with total scores ranging from 7 to 9. Two studies—Henderson (17) and Hendrix (27)—received moderate-risk ratings, each scoring 6/9. These ratings were primarily driven by limitations in the comparability domain, including baseline imbalances between exposed and non-exposed groups, along with incomplete or inadequately reported follow-up. Such limitations reduced confidence in the accuracy and certainty of outcome ascertainment compared with the higher-scoring cohort studies (Figure 3).

**Figure 2:**
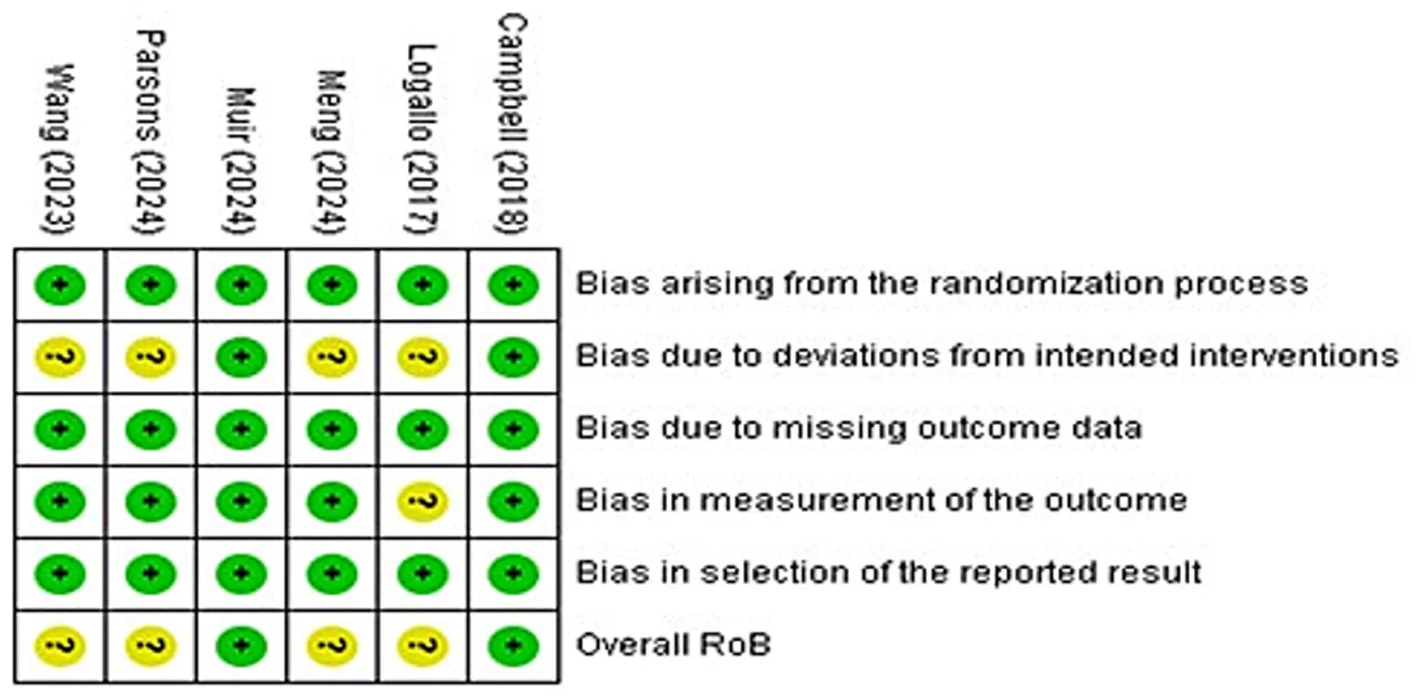
Risk of bias for RCTs.

**Figure 3:**
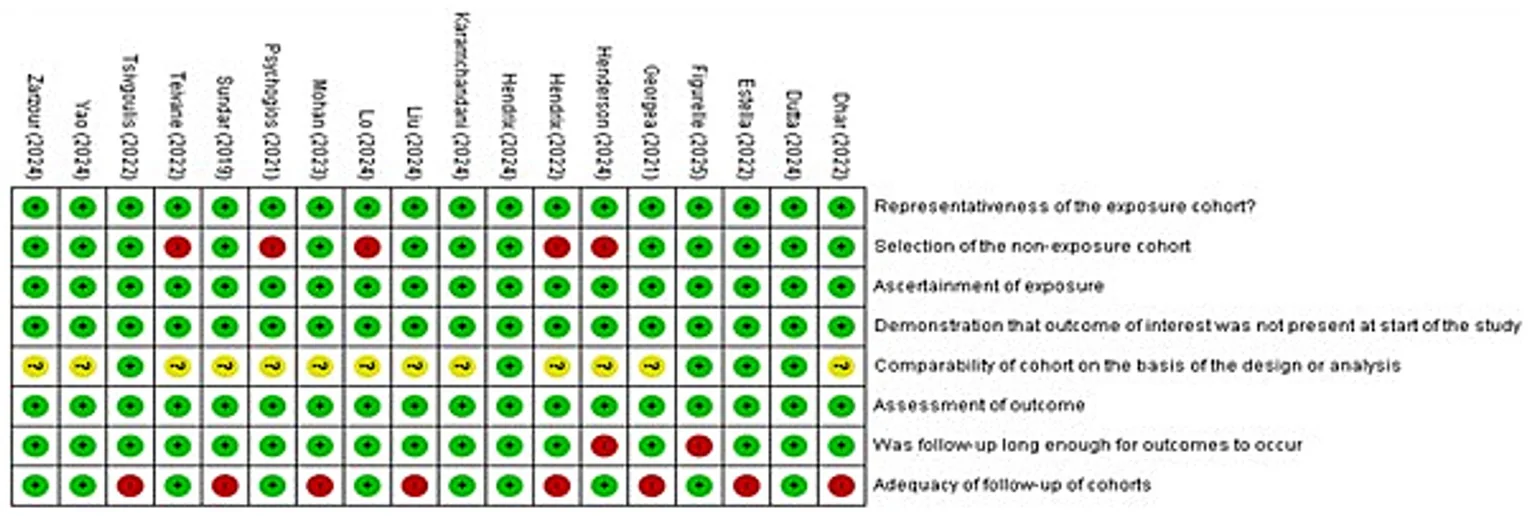
NOS score for cohort studies.

### 3.4 Certainty of evidence (GRADE assessment of outcomes)

Table 2 summarizes the certainty of evidence for all critical and important outcomes that was assessed by the GRADE approach as shown in. Outcomes mRS (0–1), sICH, mortality rates, and rate of incidence of angioedema were found to have a high certainty of evidence indicating that the effect estimates are very likely to be close to the true effect. On the other hand, the certainty of evidence was deemed moderate for several other outcomes, including rates of achieving mRS (6), improvement of mRS more than 4, mean mRS at 3 months, NIHSS at 24 h, and rate of parenchymal hematoma. These outcomes were downgraded to one level mainly due to either a serious risk of bias in some of the studies used or concerns about inconsistency due to the unexplained heterogeneity between the studies. The mRS (0–2) was considered to have low certainty of evidence due to the presence of both serious risk of bias and substantial statistical heterogeneity.

**Table 2:** Certainty of evidence.

Outcomes	Anticipated absolute effects^*^ (95% CI)		Relative effect (95% CI)	Certainty of the evidence (GRADE)
	Risk with tPA	Risk with TNK		
mRS (0–1) follow-up: 3 months	565 per 1,000	610 per 1,000 (649 to 576)	RR 1.08 (1.02 to 1.15)	⨁⨁⨁⨁ High^a^
mRS (0–2) follow-up: 3 months	654 per 1,000	674 per 1,000 (720 to 635)	RR 1.03 (0.97 to 1.10)	⨁⨁◯◯ Low^b^^,^^c^
mRS (6) follow-up: 3 months	109 per 1,000	115 per 1,000 (142 to 93)	RR 1.06 (0.86 to 1.31)	⨁⨁⨁◯ Moderate^b^
Improvement of NIHSS ≥ 4 follow-up: 3 months	429 per 1,000	488 per 1,000 (616 to 363)	OR 1.27 (0.76 to 2.14)	⨁⨁⨁◯ Moderate^d^
Mean mRS follow-up: 3 months	The mean mRs was **0**	MD 0.24 lower (0.72 lower to 0.25 higher)	-	⨁⨁⨁◯ Moderate^d^
NIHSS follow-up: 1 days	The mean NIHSS was **0**	MD 0.6 lower (1.2 lower to 0.01 higher)	-	⨁⨁⨁◯ Moderate^b^
sICH follow-up: 3 months	37 per 1,000	36 per 1,000 (44 to 30)	OR 0.99 (0.82 to 1.20)	⨁⨁⨁⨁ High
Mortality rate follow-up: 3 months	95 per 1,000	86 per 1,000 (97 to 76)	OR 0.89 (0.78 to 1.02)	⨁⨁⨁⨁ High
Angioedema follow-up: 3 months	3 per 1,000	4 per 1,000 (8 to 2)	OR 1.25 (0.60 to 2.60)	⨁⨁⨁⨁ High
Parenchymal hematoma follow-up: 3 months	62 per 1,000	59 per 1,000 (70 to 49)	OR 0.95 (0.78 to 1.15)	⨁⨁⨁◯ Moderate^b^

a There was evidence of significant statistical heterogeneity across the included studies (I2 > 50%). However this inconsistency was attributed to the difference in study design (RCT vs Cohort).

b Evidence was downgraded one level for serious risk of bias based on limitations found across both study designs. RCTs (4 of 6) had unclear risk due to limited reporting on adherence to protocols (Meng, Parsons) and lack of clear assessor blinding (Logallo). Cohort studies (17, 27) were rated moderate-risk due to limitations in the comparability domain (baseline imbalances and inadequate follow-up).

c There was evidence of substantial statistical heterogeneity across the included studies (I^2^ > 50%). This inconsistency was only present between cohort studies and was not fully explained by pre-specified subgroup analyses (e.g., dosage or patient age).

d There was evidence of substantial statistical heterogeneity across the included studies (I^2^ > 50%). This inconsistency was not fully explained by pre-specified subgroup analyses (e.g., dosage or patient age).

### 3.5 Outcomes

Table 3 summarizes the outcomes of the meta-analysis whether related to efficacy or safety endpoints.

**Table 3:** Summary of outcomes.

Outcomes		Number of patients (TNK/tPA)	Heterogeneity		Model used (Fixed/Random)	Effect size		
			*p*	I^2^ (%)		MD//OR	95% CI	*p*
Primary	mRS (0–1)	4307/5903	0.007	66	Random	1.08	[1.01, 1.15]	0.02*
	mRS (0–2)	4757/6547	< 0.00001	77	Random	1.03	[0.97, 1.10]	0.30
	mRS (6)	1353/1371	0.28	21	Fixed	1.06	[0.86, 1.31]	0.57
	NIHSS improvement ≥ 4	1946/3157	< 0.00001	93	Random	1.27	[0.76, 2.14]	0.36
	Mean mRS at 3 months	2716/5133	< 0.00001	93	Random	−0.24	[−0.72, 0.25]	0.34
	Mean NIHSS at 24 h	2874/4259	0.002	69	Random	−0.60	[−1.20, 0.01]	0.05
	Onset-to-needle time	1028/1089	0.45	0	Fixed	−3.78	[−8.71, 1.14]	0.13
	Door-to-needle time	4593/6961	< 0.00001	94	Random	−4.20	[−7.77, −0.64]	0.02*
Secondary	sICH	6073/8591	0.74	0	Fixed	0.99	[0.82, 1.20]	0.94
	Mortality rate	5442/7677	0.07	36	Fixed	0.83	[0.73, 0.94]	0.004*
	Angioedema	3308/4437	0.75	0	Fixed	1.25	[0.60, 2.60]	0.55
	Parenchymal hematoma	4019/6041	0.45	10	Fixed	0.95	[0.78, 1.15]	0.59

*p – value is significant. TNK; Tenecteplase, tPA; Tissue plasminogen activator, MD; Mean difference, OR; Odds ratio; CI; Confidence interval, mRS; Modified Rankin scale, NIHSS; National Institutes of Health Stroke Scale, sICH; Symptomatic intracranial hemorrhage.

### 3.6 Primary outcomes

#### 3.6.1 mRS

In this meta-analysis, the likelihood of achieving an mRS (0–1) was significantly higher in the TNK group compared to the tPA group (Figure 4A, RR = 1.08; 95% CI: 1.01–1.15; *p* = 0.02). Conversely, no significant difference was found in the overall improvement of an mRS (0–2) between the TNK and tPA groups (Figure 4B, RR = 1.03; 95% CI: 0.97–1.10; *p* = 0.30). Similarly, there was no significant difference in the overall improvement of an mRS (6) (Figure 4C, RR = 1.07; 95% CI: 0.84–1.36; *p* = 0.58). Finally, the mean mRS at 3 months also showed no significant difference between the TNK and tPA groups (Figure 4D, MD = −0.24; 95% CI: −0.72 – 0.25; *p* = 0.34).

**Figure 4:**
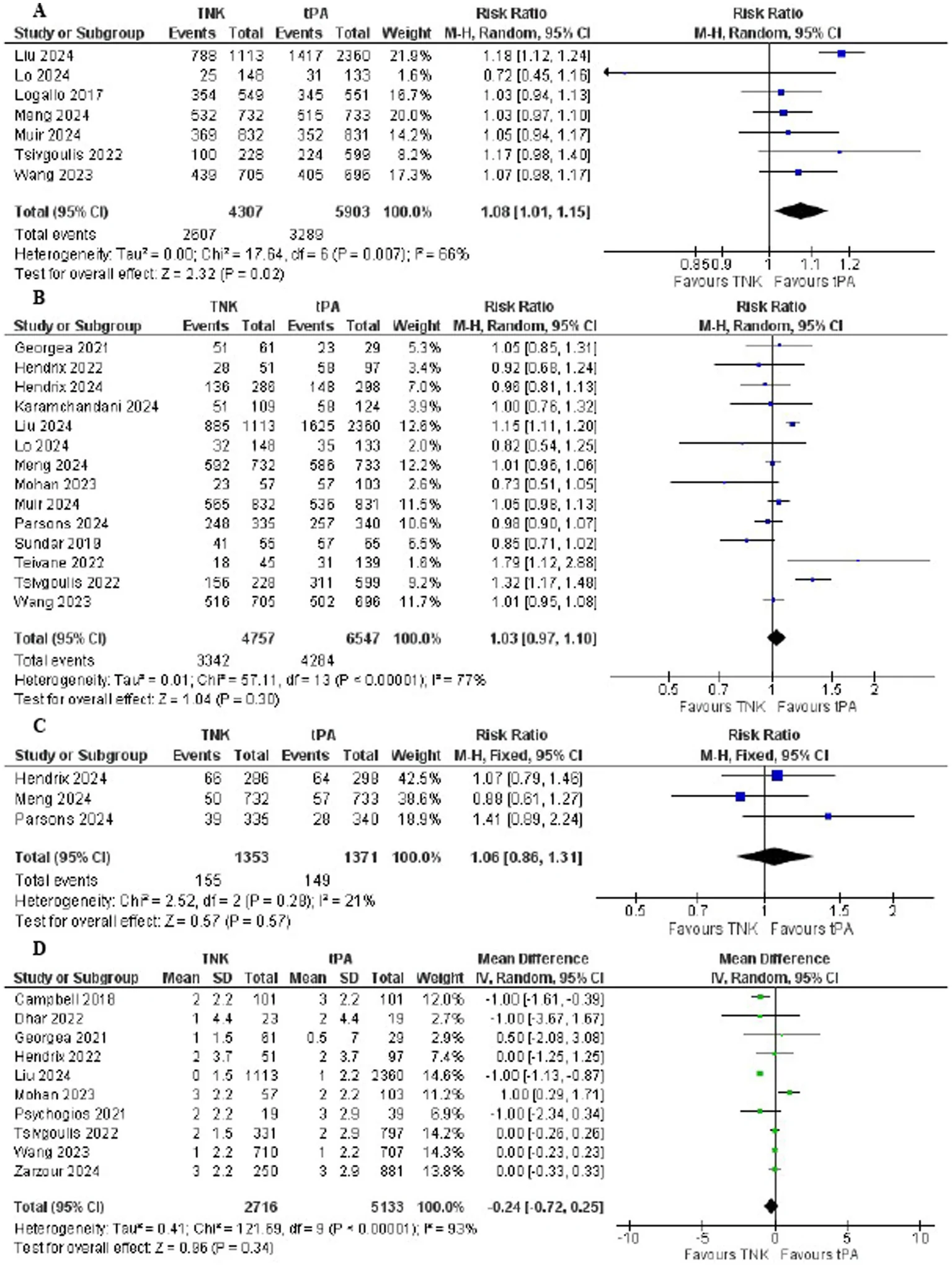
**(A)** RR of mRS (0–1). **(B)** RR of mRS (0–2). **(C)** RR of mRS (6). **(D)** MD of mRS at 3 months.

#### 3.6.2 NIHSS

There was no significant difference in the rates of improvement of the NIHSS of more than 4 points between the TNK and tPA groups (Figure 5A, OR = 1.27; 95% CI; 0.76–2.14, *p* = 0.36). Similarly, there was no significant difference in the mean NIHSS 24 h post-treatment in the TNK group when compared to the tPA group (Figure 5B, MD = −0.60; 95% CI; −1.20 – 0.01, *p* = 0.05).

**Figure 5:**
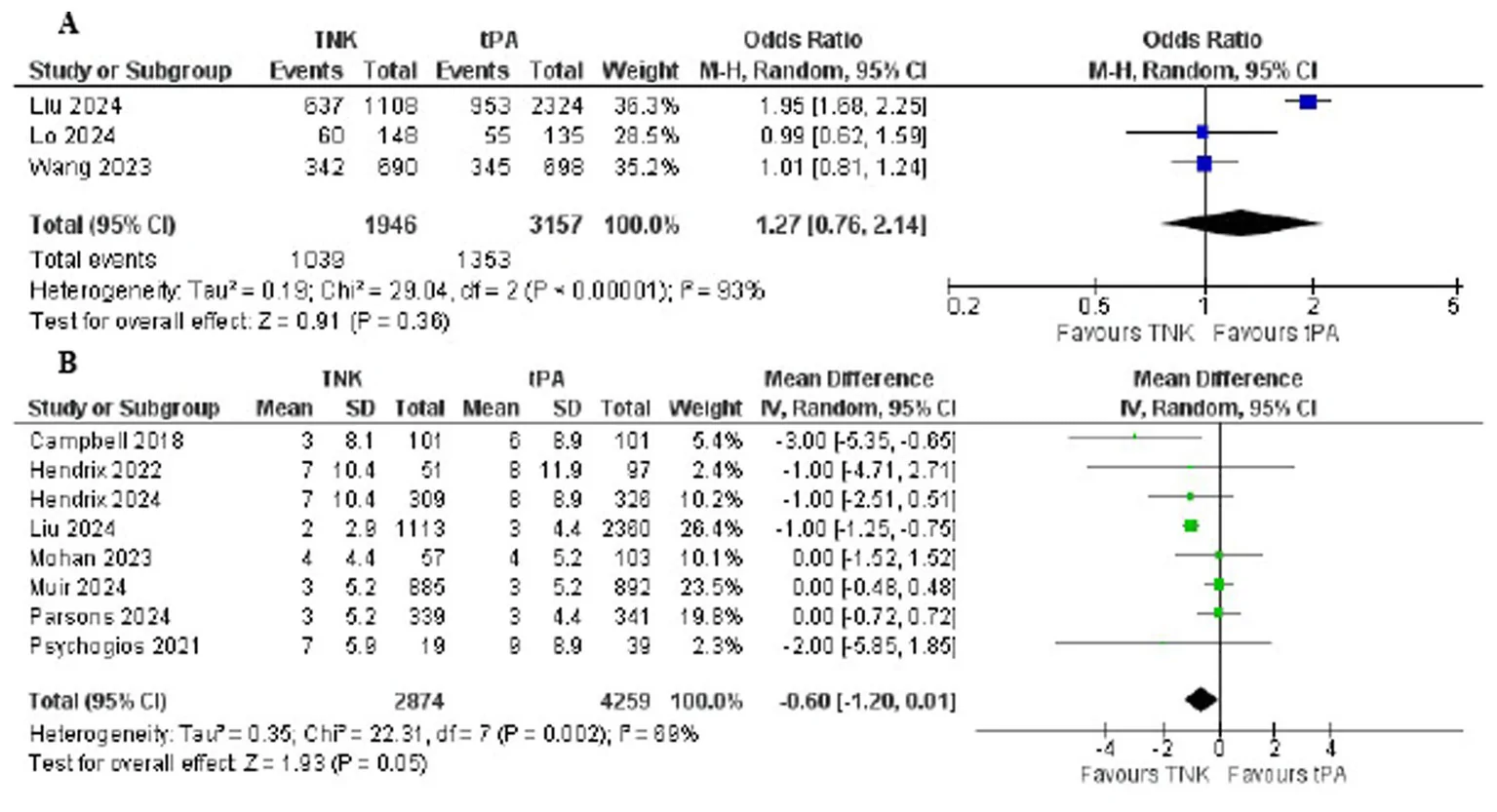
**(A)** OR of NIHSS of more than 4 points. **(B)** MD of mean NIHSS 24 h post-treatment.

#### 3.6.3 Duration of intervention

There was no significant difference in the OTN time in the TNK group compared to the tPA group (Figure 6A, MD = −3.78; 95% CI; −8.71 – 1.14, *p* = 0.13), however, TNK had significantly faster DTN time compared to the tPA group (Figure 6B, MD = −4.20; 95% CI; −7.77 – 0.64, *p* = 0.02).

**Figure 6:**
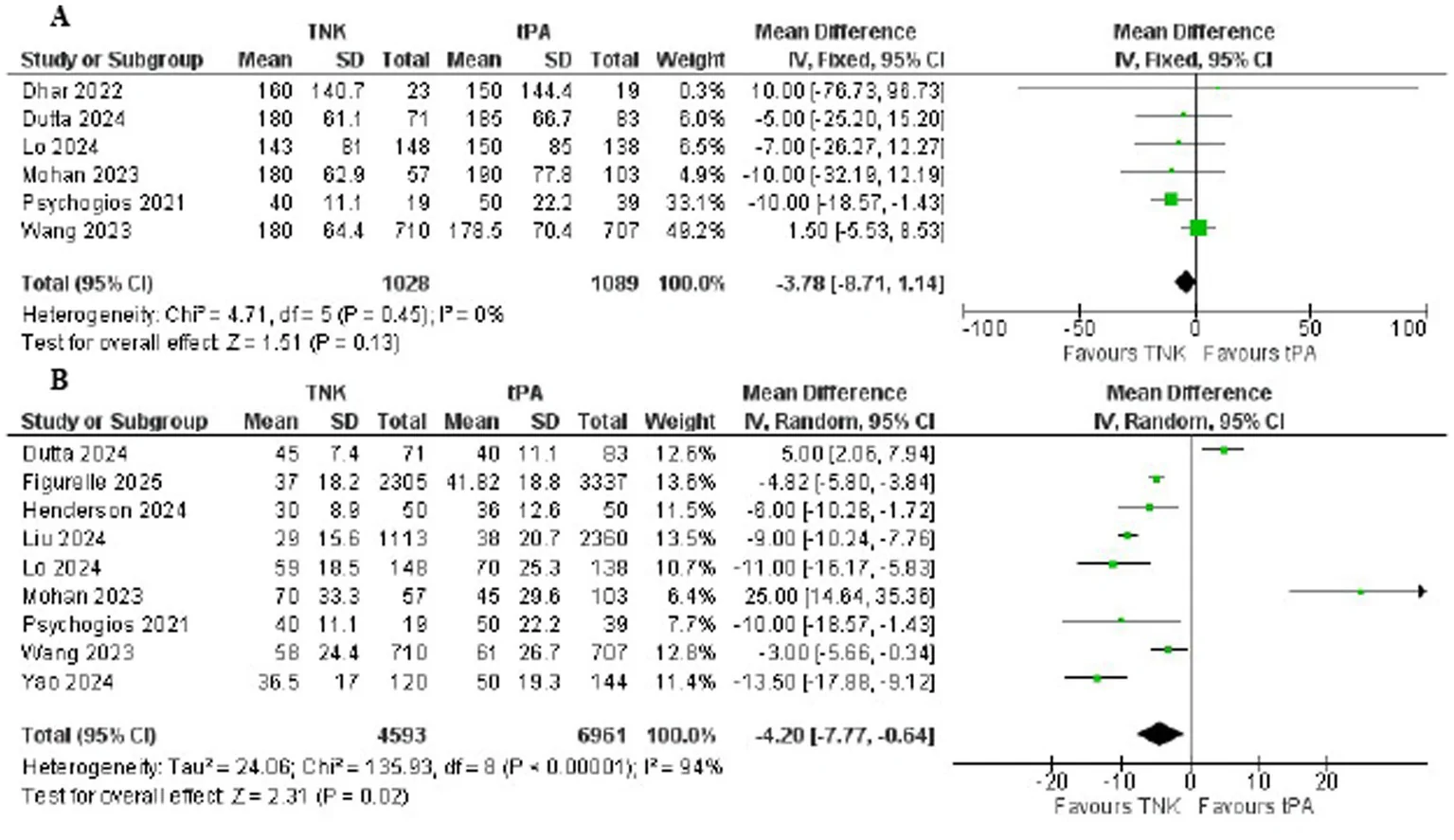
**(A)** MD of mean onset-to-needle time. **(B)** MD of mean door-to-needle time.

### 3.7 Secondary outcomes

#### 3.7.1 Bleeding

There was no significant difference in the rates of sICH in the TNK group compared to the tPA group (Figure 7A, OR = 0.99; 95% CI; 0.82–1.20, *p* = 0.94). This was also the case when parenchymal hemorrhage was considered (Figure 7B, OR = 0.95; 95% CI; 0.78–1.15, *p* = 0.59).

**Figure 7:**
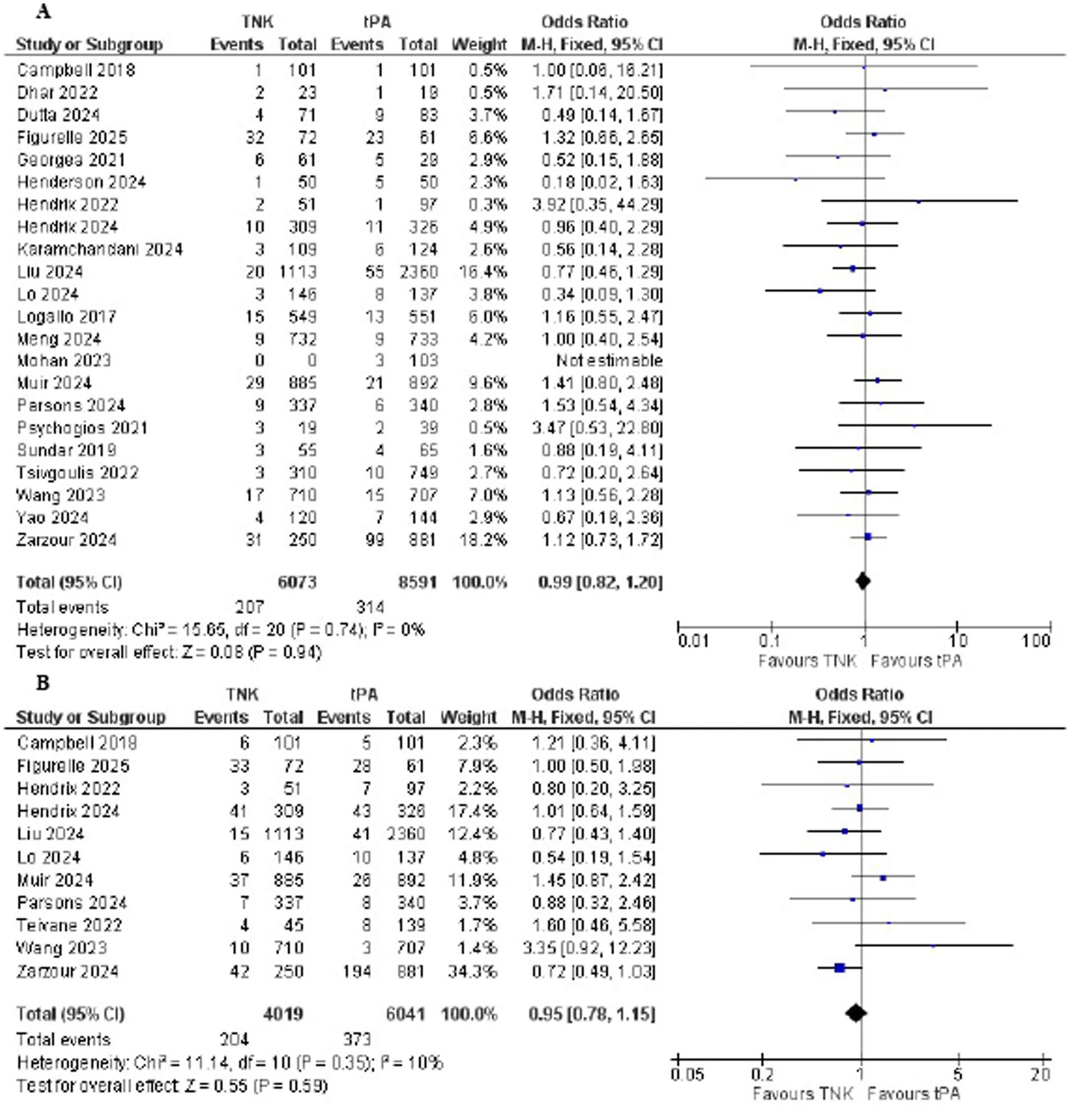
**(A)** OR of sICH. **(B)** OR of parenchymal hemorrhage.

#### 3.7.2 Mortality rate

The mortality rate was significantly less in the TNK group compared to the tPA group (Figure 8, OR = 0.83; 95% CI; 0.73–0.94, *p* = 0.004).

**Figure 8:**
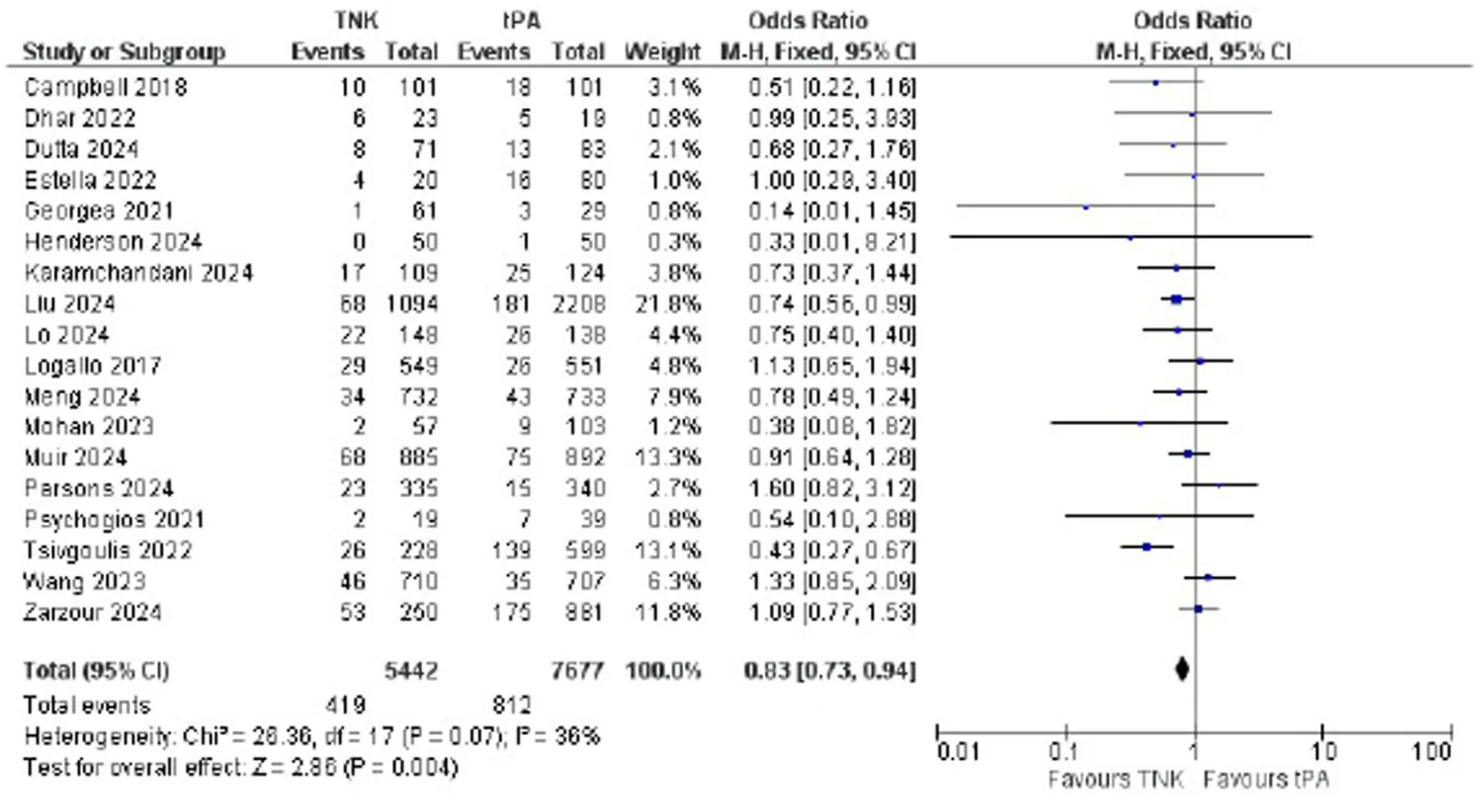
OR of mortality rate.

#### 3.7.3 Angioedema

There was no significant difference in the incidence of angioedema in the TNK group compared to the tPA group (Figure 9, OR = 1.25; 95% CI; 0.60–2.60, *p* = 0.55).

**Figure 9:**
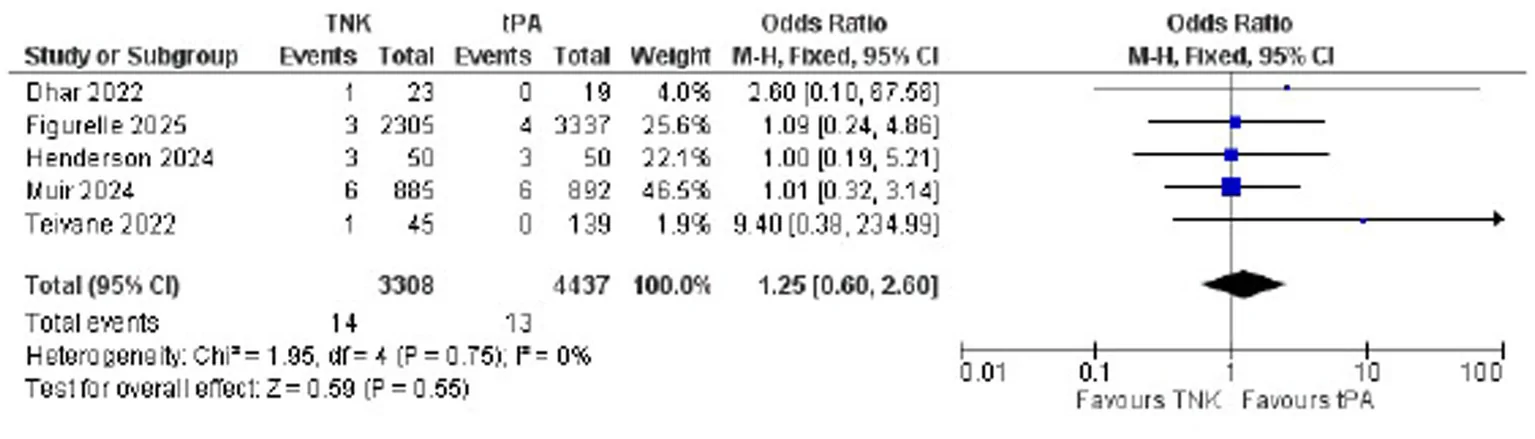
OR of angioedema.

### 3.8 Subgroup analysis

The main outcomes that were eligible to be analyzed based on study design were; the rate of an mRS score (0–1), (0–2), mean mRS at 3 months, and the NIHSS after 24 h. Our analysis revealed that there was no heterogeneity between the studies following an RCTs for the mRS score (0–1), (0–2) (Supplementary Figures S3, S4; respectively). No change of heterogeneity was noticed in the mean mRS at 3 months, and the NIHSS after 24 h (Supplementary Figures S5, S6; respectively). Despite this, our analysis revealed that there was no significant difference between the subgroups for all four outcomes (*p* = 0.29, *p* = 0.79, *p* = 0.17, *p* = 0.61; respectively). Similarly, the outcomes that were eligible to be analyzed based on TNK dose were; the rate of an mRS score (0–2), and mean mRS at 3 months. The dose used for the subgroups were 0.25 mg/kg and 0.2 mg/kg. Our analysis revealed no significant difference between the subgroups for both outcomes (Supplementary Figure S7; *p* = 0.32, Supplementary Figure S8; *p* = 0.99; respectively).

Subgroup analysis was performed based on study design to clearly report the contribution of observational studies to the results. Eligible outcomes included: sICH, and mortality rates. No significant difference was found between the RCTs and cohort groups for sICH rates (Supplementary Figure S9; *p* = 0.10). However, for mortality rates, studies following an RCT protocol showed no significant difference in the mortality rates between TNK and tPA groups, which is contrary to the overall result (Supplementary Figure S10, OR = 0.99; 95% CI; 0.82–1.21, *p* = 0.95).

### 3.9 Sensitivity analysis

A leave-one-out sensitivity analysis was performed for all our outcomes to see the effect of removing studies on the overall effect estimates and heterogeneity. Lo 2024 (18) appeared to be the cause of heterogeneity between cohort studies for achieving mRS (0–1); and caused the result to significantly favor the alteplase group (Supplementary Figure S11). Similarly, Liu 2024 (24) was found to be the cause of heterogeneity in the cohort group for the mean mRS after 3 months, decreasing it from 92 to 41%; *p* = 0.12 (Supplementary Figure S12). This was also the case for the rate of improvement by ≥ 4 points for NIHSS where the overall heterogeneity dropped from 93 to 0%; *p* = 0.96 (Supplementary Figure S13). Lastly, removing Tsivgoulis 2022 (31) caused the overall mortality rate to be insignificant between TNK and alteplase (Supplementary Figure S14, OR = 0.89; 95% CI; 0.78–1.02, *p* = 0.09).

### 3.10 Publication bias

Due to enough studies, funnel plots were evaluated for only some outcomes including sICH, parenchymal hemorrhage, and mortality rates, all of which showed no asymmetry. This reflects on a lower probability of publication bias to be present (Figure 10).

**Figure 10:**
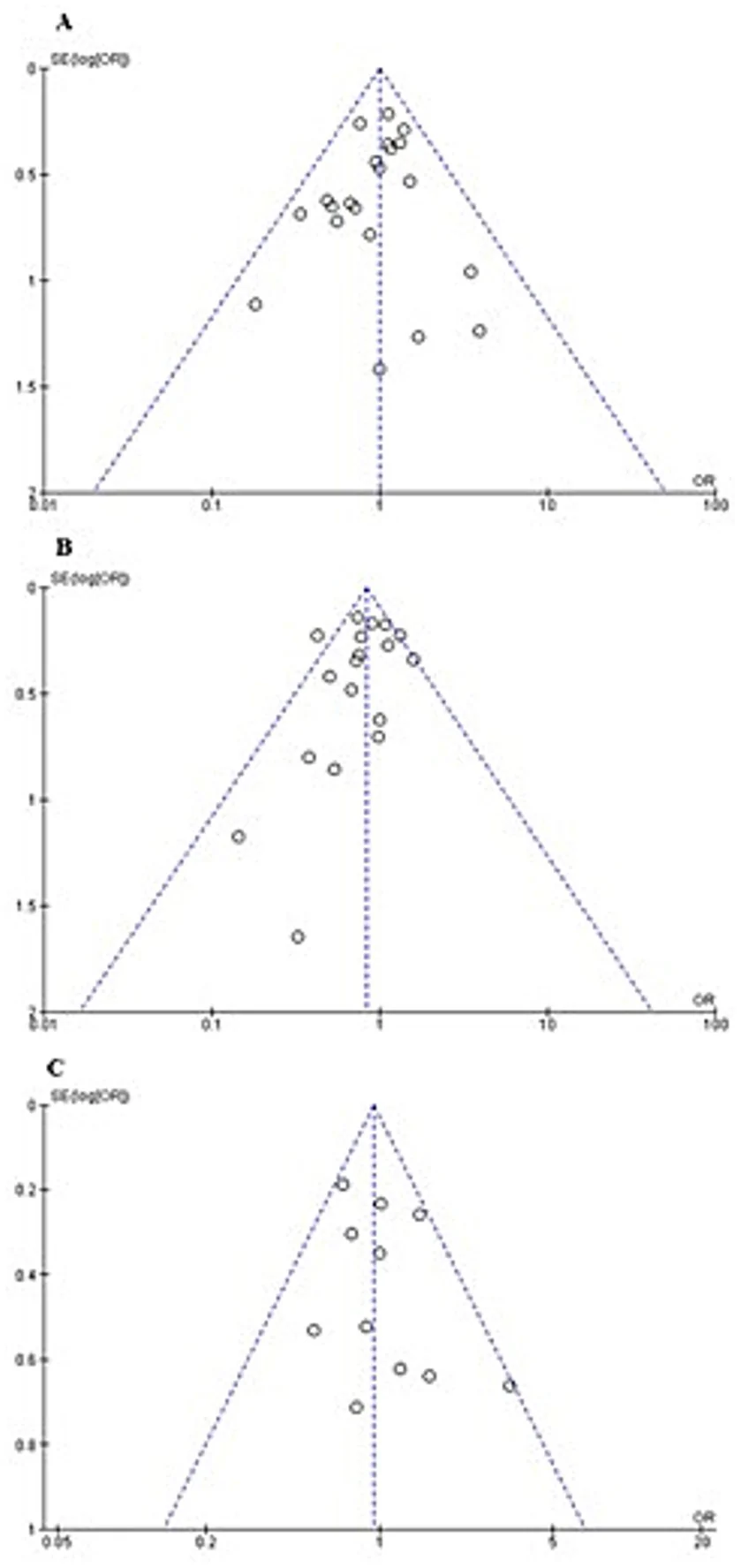
**(A)** sICH. **(B)** Parenchymal hemorrhage. **(C)** Mortality rate.

## 4 Discussion

In the present analysis, there was no significant difference in the mean NIHSS score or the rate of improvement 24 h after treatment with tenecteplase compared with tPA. Additionally, our subgroup analysis of RCTs only showed no significant difference between TNK and tPA in NIHSS scores at 24 h (*p* = 0.45). This is supported by evidence from various trials, most notably the ORIGINAL trial, which revealed that the rates of early, significant improvement in NIHSS scores, defined as a four-point improvement from NIHSS at presentation or an NIHSS score of zero at 24 h post-treatment, were slightly higher in the tenecteplase arm but not significantly different between the two drugs (38). The EXTEND-IA TNK trial demonstrated a trend toward greater improvement in NIHSS scores at 24 h with tenecteplase compared to alteplase. Still, this difference fell short of statistical significance (*p* = 0.06) (36). Similarly, while a recent meta-analysis of nine RCTs showed greater early neurological improvement with tenecteplase versus alteplase, this difference was not statistically significant and was limited by substantial heterogeneity (40). Although a pooled analysis from the Australian-TNK and ATTEST trials found that participants with complete vessel occlusion receiving tenecteplase had significantly more improvement in NIHSS scores at 24 h, subgroup analysis revealed that this effect was mainly observed in patients with large vessel occlusion and thus may not be generalisable (41). Furthermore, the follow-up ATTEST-2 trial did not report a statistically significant difference between tenecteplase and alteplase (21). In some of these studies, the potential early neurological improvement observed with tenecteplase may be explained by its longer half-life and potent clot dissolution, resulting in more rapid recanalization (42).

Patients receiving TNK were statistically more likely to achieve an excellent functional outcome (mRS score 0–1) at 90 days than patients receiving tPA, indicating better long-term outcomes in the tenecteplase arm in this analysis. In support of this are results from an extensive retrospective multicenter study using data from the Effectiveness of Routine Tenecteplase vs. Alteplase in Acute Ischemic Stroke (CERTAIN) collaboration, which reported higher odds of achieving an excellent functional outcome with tenecteplase compared to alteplase (43). Moreover, in a pre-specified analysis of the AcT trial on patients with large vessel stroke who did not undergo thrombectomy, the rates of achieving an excellent functional outcome were higher in patients receiving tenecteplase after adjusting for confounders (44). In our pooled analysis of RCTs, TNK was associated with a higher rate of excellent functional outcome (mRS 0–1) at 3 months compared with alteplase (tPA) (RR ≈ 1.04, 95% CI:1.00–1.09; *p* = 0.04), with no significant heterogeneity across studies. A prior meta-analysis of RCTs failed to demonstrate a significant difference in functional outcomes between tenecteplase and alteplase (45). Previous meta-analysis also did not show a difference in rates of mRS scores <1 for tenecteplase and alteplase at 90 days (40, 46, 47). However, Singh et al. noted significant heterogeneity and suspected publication bias in their study (40). These findings suggest that while earlier analyses did not demonstrate a clear advantage of tenecteplase over alteplase, recent studies increasingly support a potential benefit. Although a retrospective analysis using data from the Safe Implementation of Treatments in Stroke (SITS) International Register found that the odds of achieving an mRS score of 0–2 were higher in those treated with tenecteplase, our study found no significant difference between the two medications which is supported by the results of several previous meta-analyses (31, 40, 46, 47). Overall, results from the CERTAIN collaboration revealed that tenecteplase was associated with a 25% reduction in the odds of a higher mRS score at 90 days, specifically, 29% lower odds of mRS three or greater compared to alteplase (43). Nonetheless, our study found no significant difference in the mRs at 90 days, but this is limited by substantial heterogeneity. These discordant results may be due to variations in study designs, population studies, or tenecteplase doses studied.

In this analysis, the OTN time was not significantly different between the tenecteplase and alteplase groups. Results from prior observational studies and RCTs are conflicting regarding the time from symptom onset to the initiation of intravenous thrombolysis. Multiple studies, including the seminal AcT trial, reported similar onset to needle times with tenecteplase and tPA (30, 48, 49). A single-center recent survey revealed that tenecteplase was associated with a significant 30-min decrease in onset to needle time in the tenecteplase group compared to the alteplase group (133.00 vs. 163.72 min) (26). Another study conducted during the COVID-19 pandemic demonstrated a significant decrease in onset to needle time in patients receiving tenecteplase (50). On the other hand, tenecteplase was significantly associated with longer OTN time in an analysis using data from the SITS International Register (31). Differences in onset-to-needle time findings in these studies may be attributed to variations in pre- and in-hospital workflows.

Regarding DTN, the TNK group had a significantly shorter DTN time than the tPA group, which is consistent with results from prior research (26, 44, 51–54). In a prospective cohort study, 41% of the patients receiving tenecteplase met the target DTN of under 45 min compared with only 29% of patients receiving alteplase (52). The AcT trial was alone in reporting no difference in door-to-needle times between the two medications (48). This finding may be attributed to the nature of RCTs, wherein strict protocols and standardized workflows minimize variability in the treatment processes, thus reducing the time advantage of tenecteplase as a single bolus.

Our pooled analysis of RCTs only in the sICH revealed no significant difference (*p* = 0.20). In line with findings from prior meta-analyses, there was no significant difference in the rates of sICH and parenchymal hemorrhage between tenecteplase and alteplase (55, 56). Similar results were reported across several randomized trials, including ATTEST 2, EXTEND-IA TNK, ORIGINAL, and a pre-specified analysis of the AcT trial for patients with large vessel occlusion (10, 21, 38, 44). Notably, one previous meta-analysis of RCTs demonstrated significantly higher sICH rates in patients receiving higher doses of tenecteplase (0.4 mg/kg) than alteplase (57). In contrast, a retrospective analysis from the CERTAIN collaboration reported significantly lower rates of sICH with tenecteplase compared to alteplase and no difference in the rates of sICH between patients receiving the 0.25 mg/kg dose and those receiving the 0.40 mg/kg dose (43). Differences between studies may partly be explained by using varying definitions of sICH, each of which applies distinct clinical and radiological thresholds (58–60).

The ORIGINAL and the ATTEST-2 trials did not demonstrate an advantage for TNK over tPA in terms of mortality rates (21, 38). The AcT trial also failed to show a mortality benefit with tenecteplase over alteplase in patients with large vessel occlusions (6). The results from the EXTEND-IA TNK did not reach statistical significance (10). This finding is consistent with the results of our subgroup analysis that only included RCTs (*p* = 0.95). A recent meta-analysis demonstrated that evidence from RCTs found comparable all-cause mortality rates, whereas analysis from non-randomized studies suggested lower mortality with tenecteplase (55). Our analysis, including RCTs and non-randomized studies, demonstrated lower mortality in the tenecteplase group. Other meta-analyses suggested no difference in mortality rates between alteplase and tenecteplase (46, 47). Only a post-hoc analysis of the NOR-TEST trial, in which patients received the 0.4 mg/kg TNK dose, found higher mortality rates with tenecteplase but only in severe stroke patients (11). These differences likely reflect variations in study design (randomized or non-randomized studies), differences in the tenecteplase dose used, ranging from 0.1 mg/kg to 0.4 mg/kg, and differences in stroke severity and location among study populations.

As with previous trials and observational studies, no significant difference in angioedema or parenchyma hemorrhage rates between TNK and tPA was noted (38, 54). The FDA drug label for tenecteplase cites a 1.0% risk of developing orolingual angioedema in patients receiving tenecteplase (61). Tenecteplase catalyzes the conversion of plasminogen to plasmin, which subsequently activates the kallikrein–kinin system, increasing bradykinin levels. Bradykinin, in turn, causes vasodilation and increased vascular permeability, resulting in angioedema (62). Although specific risk factors for tenecteplase-associated angioedema are not well defined in the literature, they are likely similar to those reported for alteplase, given their shared mechanism of action. These risk factors include female sex, use of ACE inhibitors, as well as comorbidities including diabetes, hypertension, and dyslipidemia (63). A recent American Heart Association scientific statement emphasizes the need for protocols to respond to angioedema after thrombolytic therapy (64).

Clinically robust data from the pooled data indicate that TNK is non-inferior to tPA, with selective advantages in achieving excellent functional outcomes (mRS 0–1) and reducing mortality in cohorts subgroups analysis, without an associated increase in major safety events. In addition, TNK demonstrates a consistent workflow advantage, particularly in lowering door-to-needle time, which may be carefully interpreted due to high heterogeneity. These findings highlight the need for further high-quality RCTs.

Certain limitations of this analysis must be acknowledged. To begin, this study included both observational and clinical trials, and the studies utilized variable doses of tenecteplase, while three failed to report the dose. Secondly, while different sICH criteria were used including ECASS-III, SISTS-MOST and NINDS, the core clinical syndrome these criteria attempted to capture is similar. All definitions required clinically significant neurological deterioration defined as death or worsening of the NIHSS score by four or more points that correlates with radiologically confirmed hemorrhage within a maximum of 36 h from alteplase and tenecteplase administration. Third, two of the cohort studies included had a moderate risk of bias rating due to issues related to cohort comparability, inadequate follow-up, and discrepancies in the timing of inclusion between the two groups. Fourth, we could not feasibly perform dose-specific subgroup analysis for 0.4 mg/kg tenecteplase because this dose was reported in only one included study (37), preventing a meaningful pooled comparison. Fifth, lack of gray literature or trial registry. Sixth, lack of access to the Embase database may have resulted in the omission of potential eligible studies. Finally, significant heterogeneity was observed for several outcomes.

Given the rapidly evolving evidence base, future updates of this meta-analysis incorporating recently published and ongoing randomized and large real-world studies are warranted, as additional data may further refine effect estimates, particularly for functional outcomes such as mRS 0–1 and may help reduce residual heterogeneity.

## 5 Conclusion

This systematic review and meta-analysis provides evidence comparing TNK and tPA in randomized and observational studies. However, interpretation of certain efficacy outcomes—including mRS (0–2), NIHSS improvement ≥4, door-to-needle time, and mean mRS—was limited by substantial heterogeneity and should therefore be interpreted with caution. Safety profiles were generally comparable, while observational studies demonstrated a lower mortality rate with TNK compared with tPA. Overall, our findings indicate that TNK is non-inferior to tPA.

## Data Availability

The original contributions presented in the study are included in the article/Supplementary material, further inquiries can be directed to the corresponding author.
