# Supplementary material for: Tenecteplase in acute ischemic stroke: an updated meta-analysis
Source: Front Neurol. 2026 Sep 15;17:1921538. doi: 10.3389/fneur.2026.1921538 (PMC13619431; doi:10.3389/fneur.2026.1921538)
Supplement: Supplementary file 1 [file Table_1.DOCX]

Supplementary Material

**Table S1**. Search Strategy

(“minor stroke” OR “transient ischemic attack” OR “cerebral stroke” OR “cerebral infarction” OR “cerebrovascular disease” OR “cerebral ischemia” OR “cerebrovascular disorders” OR “cerebrovascular accident” OR “brain ischemia” OR “acute stroke” OR “brain infarction” OR “light stroke” OR “mild stroke” OR “silent stroke”)

**AND**

(“tenecteplase” OR “TNK-tPA” OR “TNK” OR “TNKase” OR “rhTNK-tPA”)

**AND**

(“alteplase” OR “rt-PA” OR “tissue plasminogen activator” OR “plasminogen activator tissue type”)

**AND**

(“randomized controlled trial” OR “controlled clinical trial” OR “randomized” OR “placebo-controlled” OR “drug therapy” OR “clinical trial” OR “random assignment” OR “trial” OR “study groups” OR “cohort study” OR “retrospective study” OR “prospective study” NOT (“animals” NOT “humans”)

**S2.** Definitions of the outcomes

The mRS assesses neurological recovery at 3 months. Excellent recovery is defined as a score of (0-1), good recovery is defined as a score of (0-2), and death is defined as a score of (6). The NIHSS is based on neurological improvement in each included study. Most studies defined improvement as 4-8 or more points or a return to a total score of 0 or 1 points on the NIHSS. OTN time is defined as the time interval between the beginning of the patient’s symptoms and the delivery of the thrombolytic, whereas DTN time is defined as the time elapsed between the patient’s arrival at the hospital and the administration of the thrombolytic. The definition of sICH varied between studies. The majority of studies defined sICH as any intracerebral hemorrhage, including subarachnoid hemorrhages, on follow-up imaging associated with a decline in neurological function marked by a deterioration of four points on the NIHSS score from baseline (10, 17, 24-26, 31, 36). Other studies used the Safe Implementation of Thrombolysis in Stroke- Monitoring Study (SITS-MOST) criteria, which defined sICH as a type 2 parenchymal hemorrhages on post-treatment imaging leading to death or marked by an increase of four points on the NIHSS score from baseline or the lowest NIHSS score (18, 21, 27, 29-30, 32, 35). Some studies used the European Cooperative Acute Stroke Study (ECASS-3) criteria, defined as death or neurological deterioration of 4 points on the NIHSS score associated with any extravascular blood within the cranium (23, 28, 38-40). One study used similar ECASS-2 criteria (34). Two studies did not clearly define what constitutes sICH (19, 22), and one study did not differentiate between symptomatic and asymptomatic ICH (33).

**Figure S3**. mRS (0-1) RCTs vs Cohort

**Figure S4**. mRS (0-2) RCTs vs Cohort

**Figure S5**. NIHSS after 24 hours RCTs vs Cohort

**Figure S6**. Mean mRS at 3 months RCTs vs Cohort

**Figure S7**. mRS (0-2) 0.2 vs 0.25 mg/kg

**Figure S8**. Mean mRS at 3 months 0.2 vs 0.25 mg/kg

**Figure S9**. sICH rates RCTs vs Cohort

**Figure S10**. Mortality rate RCTs vs Cohort

**Figure S11**. mRS (0-1) RCTs vs Cohort (Lo 2024)

**Figure S12**. Mean mRS at 3 months RCTs vs Cohort (Liu 2024)

**Figure S13**. Rate of improvement of mRs more than 4 RCTs vs Cohort (Liu 2024)

**Figure S14**. Mortality rate RCTs vs Cohort (Tsivgoulis 2022)
